# Enzymatic Biocontrol of Fire Blight (*Erwinia amylovora*) Using an Engineered Glycosyl Hydrolase

**DOI:** 10.1021/acs.est.5c16698

**Published:** 2026-05-09

**Authors:** Kevin J. Lynn, Cole Clapper, Evan Kulp, Nathanial J. Boeckman, Matheus C. Borba, Emmanuel Sempeles, Joseph Capobianco, Srđan Aćimović, Bryan W. Berger

**Affiliations:** † University of Virginia, Charlottesville, Virginia 22903, United States; ‡ 1757 Virginia Polytechnic Institute and State University, Blacksburg, Virginia 24061-0131, United States; § Characterization and Interventions for Foodborne Pathogens, Agricultural Research Service, United States Department of Agriculture, Wyndmoor, Pennsylvania 19038, United States

**Keywords:** Fire blight, Erwinia amylovora, Antibiotic resistance, Biofilm degradation, Exopolysaccharide, Glycoside hydrolase, Biocontrol, Antimicrobial enzyme, Sustainable agriculture

## Abstract

Current management of fire blight, caused by *Erwinia amylovora,* relies heavily on streptomycin a practice that has contributed to the emergence of antibiotic-resistant strains and raised environmental and regulatory concerns. Enzyme-based biocontrol agents offer a promising antibiotic-free approach that combines target specificity with environmental compatibility. This study evaluates CAase, a bacteriophage-derived glycosyl hydrolase, for its ability to disrupt *E. amylovora* biofilms and reduce disease severity. Biochemical assays and scanning electron microscopy confirmed that CAase efficiently degraded the extracellular polysaccharide (EPS) matrix, releasing cells from biofilms. Gas chromatography–mass spectrometry (GC–MS) linkage analysis of EPS isolated from two *Erwinia amylovora* strains demonstrated that CAase preferentially cleaves galactan-rich amylovoran produced by strain EA273, while exhibiting only limited activity toward the levan-rich EPS from strain EA1430. Functional assays revealed that CAase reduced bacterial viability by nearly 2 orders of magnitude at higher enzyme concentrations, strongly suppressed surface motility, and induced ultrastructural damage visible by transmission electron microscopy. Importantly, field trials showed that CAase significantly lowered blossom and shoot blight incidence under orchard conditions. These results highlight CAase as a potent enzyme-based strategy for reducing the virulence of *E. amylovora* and demonstrate its potential as a sustainable alternative to antibiotics in fire blight management.

## Graphical abstract

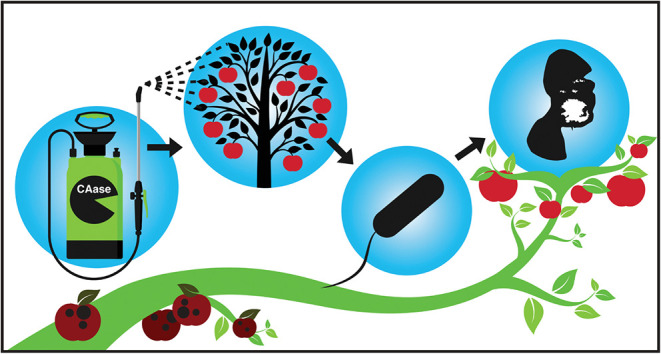

## Introduction

Fire blight, caused by the Gram-negative bacterium *Erwinia amylovora*, is a destructive disease of pome fruit trees such as apple (*Malus domestica*) and pear (*Pyrus communis*), that threatens orchard productivity and sustainability worldwide.^1^ Originally reported in North America in the 18th century, the disease has since spread to Europe, the Middle East, and other temperate fruit-producing regions.^1^ Outbreaks can decimate entire orchards, which can lead to reductions in yield and long-term economic impacts. In the United States, annual losses are estimated at $100 million while a 2007 outbreak in Switzerland caused an estimated $56 million in damage.^1,2^

Current fire blight management relies heavily on antibiotics, particularly streptomycin, which has been used in North American orchards since the 1950s to suppress *E. amylovora* during bloom.^2,3^ Although streptomycin remains a highly effective treatment, its routine use has accelerated the emergence of antibiotic-resistant strains.^4,5^ Streptomycin-resistant strains of *E. amylovora* were first reported in the 1970s and now complicate control efforts in several regions.^6^ Beyond the pathogen, frequent antibiotic applications also lead to resistance in epiphytic microbes and pose broader ecological concerns, including effects on nontarget organisms.^7–9^ Regulatory restrictions are mounting, and the European Union has banned antibiotics for agricultural use, underscoring the need for sustainable alternatives.^8^

As antibiotic resistance and regulatory constraints grow, researchers have explored antibiotic-free solutions, such as antagonistic bacteria, bacteriophages, and recombinantly produced antimicrobials.^10,11^ Among these, enzyme-based biocontrol agents are particularly promising as they offer substrate specificity, biodegradability, and a lower risk of resistance development.^5^ One such enzyme, CAase, is a phage-derived glycosyl hydrolase that degrades microbial exopolysaccharides.^12^ CAase cleaves glycosidic linkages within the extracellular polymeric substances (EPS) matrix of biofilms, which disrupts cell adhesion and biofilm stability.^13^

Multiple studies have demonstrated the efficacy of CAase against biofilm-forming pathogens on abiotic and food-associated surfaces. Mayton et al. (2021) reported that CAase reduced biofilm biomass of *Escherichia coli* (*E. coli*), *Salmonella*, and *Listeria monocytogenes* by approximately 30–40% in microplate assays on abiotic plastic surfaces and enhanced removal of mature biofilms relative to control treatments.^12^ In addition, this study demonstrated increased detachment of *E. coli* cells from spinach leaf surfaces, representing a produce-associated model system. Felton et al. (2023) showed that CAase improved detection of *L. monocytogenes* by releasing pathogens from food and food-contact surfaces.^14^ More recently, Renye et al. (2025) combined CAase with a bacteriocin to eliminate persistent *Listeria* biofilms, demonstrating synergistic activity and structural disruption of the bacterial surface.^13^ Together, these studies show CAase has versatility against many pathogens and suggest that CAase degradation of biofilm infrastructure will lead to enhanced effectiveness of companion antimicrobials.

## Materials and Methods

### Bacterial Strains

*Erwinia amylovora* strains EA273 (ATCC 49946), EA1430 (CFBP 1430), and EA110 were used in this study. EA273 was isolated in 1971 from a diseased apple in New York, USA, in the laboratory of Steven Beer (Cornell University) and is a highly virulent, widely used reference strain in fire blight research. EA1430 was isolated in 1972 from *Crataegus spp.* (hawthorn) in France and represents a well-characterized European reference strain with a fully sequenced genome; comparative analyses show >99.99% genome identity between EA1430 and EA273, supporting their use as complementary in vitro models despite differences in plasmid content and regulatory features.^15^

For field experiments, *Erwinia amylovora* strain EA110 (George Sundin, Michigan State University) was selected due to its high virulence, genetic stability, and consistent performance under orchard conditions, enabling reproducible disease pressure.^16^ EA110 is a high amylovoran-producing strain, and prior comparative studies of reference isolates report similar amylovoran production for EA110 and EA273, supporting the expectation that these strains synthesize the same virulence-associated exopolysaccharide.^17^

Recombinant CAase was produced in *Escherichia coli* BL21­(DE3) (New England Biolabs).

### Bench Top CAase Preparation

CAase was produced following protocols adapted from previously described methods.^12^ Briefly, the coding sequence for a putative *Salmonella* phage-derived hydrolase (NCBI Sequence ID: YP_004893855.1) was optimized for *E. coli* codon usage and truncated at both N- and C-termini to improve soluble expression. This truncated construct was fused to a hexahistidine tag and cloned into the pET-28a­(+) vector under control of a T7 promoter. The recombinant plasmid was transformed into *E. coli* BL21­(DE3), and transformants were selected on lysogeny broth (LB)-agar plates containing 103 μM kanamycin (Research Products International).

A single colony was used to inoculate LB broth supplemented with kanamycin (103 μM), and the culture was grown for 18 h at 37 °C with agitation at 230 rpm. The starter culture was diluted into fresh LB medium (500 mL) and incubated until the optical density at 600 nm (OD600) reached 0.6–0.8. At this point, protein expression was induced by adding isopropyl β-D-1-thiogalactopyranoside (IPTG) to a final concentration of 1 mM and reducing the incubation temperature to 18 °C and 230 rpm for 20 h. Cells were then harvested by centrifugation at 3,000 × g for 10 min at 4 °C.

For purification, cell pellets from a 1 L induced culture were resuspended in 40 mL of lysis buffer (100 mM Tris, 500 mM NaCl, 10% glycerol, 10 mM imidazole) and disrupted by sonication (Qsonica, 15 W, 20% amplitude, 40 min, 4 °C) using 20 s on/off cycles. Following lysis, insoluble material was removed by centrifugation (10 min at 15,000 × g and 4 °C), and the soluble fraction was collected. Ni-NTA affinity purification was carried out using Chelating Sepharose Fast Flow immobilized metal affinity chromatography resin (Cytiva). A gravity column was conditioned by sequential treatment with a 0.2 M nickel chloride solution, DI water, and then 10 mM imidazole. Next, clarified lysate was applied, bound protein was first washed with 12 mL of 20 mM imidazole to remove nontarget proteins and then eluted with 10 mL of 300 mM imidazole to recover CAase. Elution fractions were analyzed by SDS-PAGE to assess purity.

To prepare samples for SDS-PAGE, eluted fractions were mixed with SDS loading buffer (62.5 mM Tris-HCl, 25% glycerol, 2% SDS, 0.01% bromophenol blue) and heated at 90 °C for 10 min. Proteins were separated on a 4% stacking and 12% resolving gel using a Mini-PROTEAN Tetra Cell electrophoresis system powered by a PowerPac Universal Power Supply (Bio-Rad) at 110 V for 20 min, followed by 150 V for 50 min. The gel was stained with Coomassie blue for 2 h and then destained using an ethanol/acetic acid/water (100:75:825, v/v/v) solution. The SDS gel was then imaged on an Amersham Imager 680 (Cytiva) to evaluate purity. The elution containing CAase was dialyzed against Phosphate-Buffered Saline (PBS) (pH 7.4) using a 10,000 MWCO membrane (Thermo Fisher Scientific, SnakeSkin Dialysis Tubing) for 24 h, after which it was filtered through a sterilized 0.22 μm PVDF syringe filter (Sigma-Aldrich). Protein concentrations were subsequently measured by UV absorbance (Thermo Fisher Scientific, NanoDrop 1000 Spectrophotometer) at 280 nm, using a calculated extinction coefficient of 121,990 M^–1^cm^–1^.

### Pilot Scale CAase Preparation

A 1000 L bioreactor (Eppendorf, BioFlo 720) was filled with 700 L LB (20 g/L; Research Products International), supplemented with kanamycin (85.8 μM; Research Products International, CAS 25389-94-0), then inoculated with a 7 L BL21­(DE3)/pET-28a-CAase seed culture (OD_600_ = 8.1). The culture was grown at 37 °C to OD_600_ = 0.818, cooled to 18 °C, and induced with IPTG (1 mM; Sigma-Aldrich, CAS 367-93-1) for 15 h, then chilled (<15 °C) and harvested by semicontinuous centrifugation. Frozen cell paste (−20 °C) was resuspended 1:4 (w/v) in 50 mM Tris buffer, pH 8.0 (Sigma-Aldrich, CAS 77-86-1), and lysed using a homogenizer (GEA Niro Soavi, Pony NS2006L), clarified by centrifugation (10,000 × g, 30 min), and polished using a 500 kDa hollow-fiber module (Sartorius, WA50010RES24S0, 0.5 mm lumen). The lysate was concentrated to ∼4 L with a 10 kDa poly­(ether sulfone) filter cassette (Sartorius, 3021463907E-SW) and diafiltered once with PBS (pH 7.4). Retentate (3.7 L) was stored at 4 °C, split into four sterile containers, and each brought to 3.75 L with PBS. Concentration was determined via sampling of the final product, then performing the purification and spectrophotometric techniques described in the “Bench Top CAase Preparation” section.

### Extraction and Purification of Exopolysaccharides

*Erwinia amylovora* (EA1430 and EA273) was first grown on 120 mm sucrose dense agar (SDA) plates upside down at 28 °C for 48 h. The cell mass was harvested by gently scraping the plate, suspending in 2 mL of 0.85% (w/v) NaCl, and vortexed for 20 min. The resulting suspension was centrifuged at 10,000 × g for 15 min at 4 °C, and the supernatant (containing the extracellular polymeric substances) was carefully collected. To precipitate the EPS, the supernatant was mixed with ethanol to a final ratio of (1:4) supernatant to ethanol and incubated at −20 °C for 1 h. The precipitated material was then recovered by brief centrifugation (e.g., 10 min at 10,000 × g) and washed three times with 100% ethanol (−20 °C) to remove contaminants before being air-dried for subsequent steps.

The dried precipitate was resuspended in 2 mL of a buffer comprising 1 mM CaCl_2_, 2 mM MgCl_2_, and 50 mM Tris (pH 7.5). Enzymatic treatments were carried out by adding 250 μL of 0.65 μM DNase I (New England Biolabs) and 250 μL of 2.9 μM RNase A (Thermo Fisher Scientific), followed by incubation at 37 °C for 2 h.^18^ Protein contaminants were then digested by adding 250 μL of 3.5 μM Proteinase K (Thermo Fisher Scientific) and incubating the mixture overnight at 37 °C. After enzymatic digestion, the sample was transferred into 10,000 MWCO dialysis tubing (Thermo Fisher Scientific) and dialyzed against ultrapure water at 4 °C for 24 h, with three water changes every 8 h to remove salts and other low-molecular-weight byproducts. Finally, the dialyzed EPS solution was frozen (−80 °C) and lyophilized (AAPPTec, Sharp Freeze Lyophilizer) under vacuum to yield a dry, purified EPS preparation, which was stored in a desiccator at 22 °C for subsequent analyses.

### MBTH Activity Assay

The 3-methyl-2-benzothiazolinone hydrazone (MBTH) (Sigma-Aldrich, CAS No. 149022-15-1) assay was employed to quantify the aldehyde groups on reducing sugar ends. Two solutions were prepared for this assay. Solution A contained 30 mM MBTH and 103 mM sulfamic acid (Sigma-Aldrich, CAS No. 5329-14-6) dissolved in deionized water. Solution B consisted of 38 mM iron­(III) ammonium sulfate dodecahydrate (Sigma-Aldrich, CAS No. 7783-83-7) and 103 mM sulfamic acid, also in deionized water.

To perform the assay, 50 μL of a mixture containing CAase and substrate was added to each well of a polypropylene v-bottom Multiscreen 96-well plate. Three substrates, two purified *E. amylovora* EPS (EA1430, EA273) and pectin (Sigma-Aldrich, CAS No. 9000-69-5), were tested in this study. Subsequently, 50 μL of Solution A was dispensed into each well. The plate was covered, gently agitated to ensure thorough mixing, and incubated for 1 h at 22 °C. After this incubation, 50 μL of Solution B was introduced into each well, including blank wells. The plate was then incubated for an additional 30 min at 20 °C under gentle agitation before centrifugation at 1,000 × g for 5 min and then transferring all samples to a Corning 96-well clear polystyrene microplate. Absorbance was measured at 610 nm using a BioTek Synergy Neo2 plate reader (Agilent). The 610 nm wavelength was confirmed to be the maximum absorbance by conducting the MBTH assay on 20 mM glucose and performing a spectral scan from 300 nm to 700 nm.

### Long-Term EPS Depolymerization Assay

For long-term depolymerization experiments, CAase and purified EPS were mixed on day 0 in PBS (pH 7.4) in 1.5 mL microcentrifuge tubes and protected from light. Reaction mixtures were prepared simultaneously and stored at −20 °C to synchronize experimental start times. At each designated time point (every 24 h), three replicate tubes were removed from storage, allowed to thaw, and incubated at 23 °C in the dark under gentle continuous mixing on a laboratory tube rocker (Roto-Shake Genie) operated at low speed (3) to prevent sedimentation.

Control reactions containing EPS only (no enzyme) and CAase only (no substrate) were processed in parallel and subjected to the same freeze–thaw, incubation, and mixing conditions as the EPS + CAase reactions. After a total reaction time of 200 h, all samples were analyzed for reducing ends using the MBTH assay as described above.

### Glycosyl Linkage Analysis

For CAase-treated samples, purified EPS was incubated with CAase under the same long-term depolymerization conditions used for the MBTH time-course experiments (200 h, PBS pH 7.4, 23 °C, gentle rocking) prior to recovery and preparation for GC–MS glycosyl linkage analysis.

Glycosyl linkage analysis was performed by combined gas chromatography–mass spectrometry (GC–MS) of partially methylated alditol acetate (PMAA) derivatives using a modified version of the protocol described by Willis et al. (2013) and Black et al. (2021).^19,20^ Each sample (500–1000 μg) was dissolved in approximately 300 μL of dry 1-ethyl-3-methylimidazolium acetate (Sigma-Aldrich, CAS No. 143314-17-4) and stirred overnight at room temperature. Acetylation was initiated by addition of 300 μL acetic anhydride (Sigma-Aldrich, CAS No. 108-24-7) and 50 μL *N*-methylimidazole (Sigma-Aldrich, CAS No. 616-47-7), followed by stirring for 10 min. Reactions were quenched with 1 mL ultrapure water and dialyzed (3.5 kDa MWCO dialysis tubing; Thermo Fisher Scientific, 68035) against deionized (DI) water. Samples were lyophilized, redissolved in ∼300 μL dimethyl sulfoxide (DMSO; Sigma-Aldrich, CAS No. 67-68-5), and stirred overnight.

Freshly prepared dimsyl base (∼300 μL) was added, and samples were stirred for 2 h before dropwise addition of iodomethane (∼400 μL; Sigma-Aldrich, CAS No. 74-88-4) to frozen samples, followed by an additional 2 h reaction. After extraction with water and methylene chloride (Sigma-Aldrich, CAS No. 75-09-2), the organic phase was collected, dried, and lyophilized. Uronic acid methyl esters were reduced by addition of 300 μL lithium aluminum deuteride (LiAlD_4_; AstaTech Inc., CAS No. 14128-54-2) in dry tetrahydrofuran (THF; Sigma-Aldrich, CAS No. 109-99-9) at 69 mM, followed by incubation at 80 °C for 4–6 h. Reactions were quenched with methanol:acetic acid (9:1, v/v), dried, and dialyzed (3.5 kDa MWCO) against DI water. Permethylation was performed in two rounds using sodium hydroxide (Sigma-Aldrich, CAS No. 1310-73-2) and methyl iodide with reaction times of 15 and 30 min, respectively.

Fructose-containing samples were initially hydrolyzed with 0.5 M trifluoroacetic acid (TFA; Sigma-Aldrich, CAS No. 76-05-1) at 100 °C for 1 h, followed by anomeric reduction with sodium borodeuteride (NaBD_4_; Sigma-Aldrich, CAS No. 15681-89-7) in ammonium hydroxide (Sigma-Aldrich, CAS No. 1336-21-6). Complete hydrolysis was then performed using 2 M TFA at 120 °C for 2 h, followed by a second NaBD_4_ reduction and acetylation using acetic anhydride/TFA.

Final PMAA derivatives were analyzed on an Agilent 7890A gas chromatograph coupled to a 5975C mass selective detector operating in electron-impact ionization mode, with separation achieved on a 30 m Supelco SP-2331 bonded-phase fused silica capillary column (Supelco). Glycosyl residues and linkage positions were assigned based on diagnostic electron-impact mass spectral fragmentation patterns and gas chromatographic retention behavior, using established PMAA reference libraries and retention order rules, including the University of Georgia Complex Carbohydrate Research Center (CCRC) PMAA spectral database.

### Scanning Electron Microscopy (SEM)

EA273 was cultivated in 10 mL of King’s B (KB) medium at 28 °C and 250 rpm for 18 h. After this growth period, cultures were diluted in KB to an OD_600_ of 0.8 and subsequently treated with CAase at a working concentration of 13.0 μM for 24 h or received no treatment. Twelve millimeters circle (No. 2) coverslips (Avantor, VWR^©^) were pretreated with poly-d-lysine (Thermo Fisher Scientific, CAS No. 27964-99-4) by incubating the coverslips in 1 mL poly-d-lysine solution at room temperature for 1 h, removing the excess solution, and rinsing the surface three times with deionized water. The coverslips were then allowed to air-dry, uncovered, in a biosafety cabinet.

Following pretreatment, 10 μL of each sample (either untreated or CAase-treated for 24 h) was spotted onto the dried SEM coverslips and allowed to air-dry completely (1 h). Next, 100 μL of 2.5% glutaraldehyde (Biolyst Scientific, CAS No.111-30-8) solution was applied to each coverslip and left to dry at room temperature for 5 h to fix the cells. The coverslips were immersed in phosphate-buffered saline (PBS) and stored at 4 °C prior to imaging. SEM imaging was carried out following previously described protocols, and all images were acquired on a Quanta 200 FEG scanning electron microscope (Field Electron and Ion Company).^21^

### Colony-Forming Unit Enumeration

*Erwinia amylovora* strain EA273 was revived from a −80 °C glycerol stock on KB agar and, after 24 h at 28 °C, a single colony was used to inoculate 10 mL KB broth in a 50 mL baffled flask. Cultures were grown for 18 h at 28 °C with agitation at 250 rpm, harvested by centrifugation at 1,000 × g for 5 min, and resuspended in fresh KB to an OD_600_ of 0.70 ± 0.02 (1 cm path-length). For each assay, 500 μL of this standardized suspension was combined with 500 μL of either PBS (pH 7.4) or a CAase solution prepared in PBS in sterile 4 mL polypropylene culture tubes, yielding a final volume of 1.00 mL. Tubes were incubated for a further 18 h at 28 °C, 250 rpm, after which 10-fold serial dilutions (10^0^–10^–7^) were prepared in KB. From each 10^–4^ through 10^–7^ dilution, 5 μL aliquots were spotted in triplicate onto dried KB agar plates, allowed to air-dry in a laminar-flow hood, and incubated 24 h at 28 °C. A range of dilutions was plated because countable spots (3–30 colonies) can occur at different dilution levels among samples; controls typically require the highest dilution, whereas heavily treated samples may demand lower ones. Colony counts within the countable range were averaged, multiplied by the inverse dilution factor, and divided by the plated volume to yield

$$CFU\;mL^{-1}=\frac{mean\;colony\;count}{5\times 10^{-3\;}mL}\times 10^{d}$$

where *d* is the absolute log_10_ dilution (eq 1). Biological replicates were performed for all conditions, variance among replicates was calculated, and differences between CAase-treated and control samples were assessed with an unpaired two-tailed Student’s *t* test (α = 0.05).

### Motility Assay

Motility was assessed using 0.2% agar LB Miller plates (60 mm diameter) prepared with or without CAase treatment on the surface. Each plate was spotted with a 5 μL aliquot of *Erwinia amylovora* strain EA273 adjusted to an OD_600_ of 0.6. Plates were incubated at 28 °C for 24 h under static conditions.

Following incubation, motility halos were visualized by staining the agar surface for 10 min with a 90:9.99:0.01 (v/v/v) solution of deionized water, ethanol, and crystal violet (Sigma-Aldrich, CAS No. 548-62-9). Excess stain was removed by destaining with a solution of 10% ethanol, 8% acetic acid, and 82% deionized water (v/v/v) for 60 min. Plates were imaged using an Amersham Imager 680 (Cytiva), and the area of bacterial spread was quantified using ImageJ software. Plate diameter was used to calibrate image scale for accurate area measurement.

### Cross-Sectional Transmission Electron Microscopy

Cells fixed in 2.5% glutaraldehyde were washed, postfixed with potassium ferrocyanide (Asta Tech Inc., CAS No. 13943-58-3)/osmium tetroxide (Sigma-Aldrich, CAS No. 20816-12-0), dehydrated (ethanol/acetone), and embedded in EMBed-812 resin (Biolyst Scientific). Ultrathin sections (∼70 nm) were stained with lead citrate and uranyl acetate (SPI-Chem, CAS No. 6159-44-0) and imaged using FEI Tecnai G2 F20 TEM at 120 kV.

### Plane-View Transmission Electron Microscopy

For plan-view imaging, CAase-treated and untreated cells were suspended in PBS directly applied onto carbon-coated mesh grids. Grids were then negatively stained with 1% uranyl acetate for 15 min in darkness, rinsed thoroughly with DI water, and dried before TEM imaging.

All imaging was performed using FEI Tecnai G2 F20 Transmission Electron Microscope at 120 kV.

### Field Trial

A two-year field experiment in Winchester, VA, evaluated spray treatments on 28-year-old (2023) and 29-year-old (2024) “Fuji” BC-2 apple trees, including CAase (1.82 × 10^–3^ μM and 13.0 μM), FireWall and FireLine (streptomycin sulfate 17%, oxytetracycline hydrochloride 17%; each at 16 oz/A plus Regulaid at 32 fl oz/100 gal), and Blossom Protect (Aureobasidium pullulans strains DSM 14940 and DSM 14941 at 1.25 lb/A, with Buffer Protect at 8.75 lb/A). Applications occurred at 60–70% king bloom using a tractor-carried handgun sprayer at 300 gal/A, with four trees per treatment arranged in a randomized order and buffer trees separating treatments. Flowers of cultivar “Fuji” were inoculated 24 h after treatments with *E. amylovora* strain EA110 (1 × 10^6^ CFU/mL on 4/14/2023 and 1 × 10^5^ CFU/mL on 4/15/2024, in 2 years, respectively). Both CAase and the antibiotic treatments each year were applied at 60–70% bloom open stage. Blossom Protect plus Buffer Protect was applied four times each year at Pink Bud, 20–50% bloom, 60–100% King bloom open and Petal Fall growth stages of apple flower bud development. Blossom and shoot blight incidences were rated 54 and 51 days after inoculation, respectively, by evaluating 100 randomly selected blossom clusters and shoots per tree. Percent incidence of blossom and shoot blight was calculated on a per-tree basis from 100 randomly selected clusters or shoots per tree, and treatment means were calculated from four single-tree biological replicates. Fruit russet incidence was assessed 73 and 72 days after inoculation after rating randomly selected 20 fruit clusters per tree. All statistical analyses and figure generation were performed using Microsoft Excel. Mean differences in blossom blight, shoot blight, and fruit russet incidence between treatments were analyzed by one-way ANOVA using the Excel Analysis ToolPak, followed by Tukey’s honestly significant difference (HSD) post hoc test at α = 0.05.

### Heat-Stress Susceptibility Assay

To evaluate whether CAase treatment increased susceptibility of *Erwinia amylovora* to thermal stress, a two-step enzyme–heat challenge assay was performed, adapted from established *E. amylovora* heat-stress protocols.^22^ Overnight cultures of *E. amylovora* strains EA273 and EA1430 were diluted into fresh KB medium to (OD_600_ = 0.3) and treated with either purified CAase (13.0 μM final concentration) or PBS (negative control). Cultures were incubated for 12 h at 28 °C with agitation (250 rpm).

Following enzyme treatment, biological replicates from each condition were subdivided into heated and nonheated groups. Heat stress was applied by incubating samples at 42 °C for 25 min, while nonheated controls were maintained at 23 °C. Immediately after treatment, all samples were centrifuged at 1,000 × g for 5 min, supernatants were decanted, and cell pellets were resuspended in fresh medium to a standardized optical density (OD_600_ = 0.1).

Standardized suspensions were transferred to Corning 96-well clear polystyrene microplates and incubated at 28 °C for 24 h with continuous orbital shaking. Bacterial growth was monitored by measuring OD_600_ every 15 min using a microplate reader. Growth curves were normalized to the initial OD_600_ and plotted as mean values ± standard deviation.

## Results and Discussion

### CAase Exhibits Prolonged Depolymerization Activity on *E. amylovora* EPS

To evaluate the long-term depolymerization ability of CAase, the cleavage of purified exopolysaccharides from *E. amylovora* strains EA273 and EA1430 was monitored using a time-course MBTH assay. This MBTH assay (Figure 1) quantifies aldehyde groups at newly produced reducing ends, providing a sensitive measure of glycosidic bond hydrolysis.^23^

**Figure 1:**
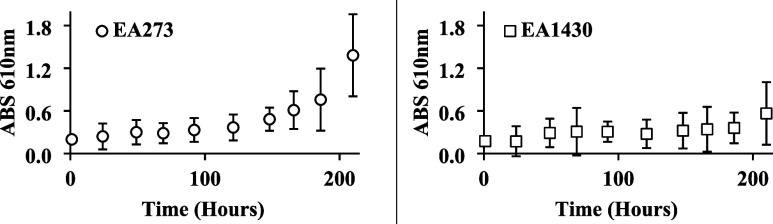
Long-term activity of CAase on EPS substrates from two *E. amylovora* strains. Left: Time-course MBTH assay of CAase (13.0 μM) activity on purified EPS from *Erwinia amylovora* EA273 (6.5 μM). Right: Time-course MBTH assay tracking cleavage of purified EPS from *E. amylovora* EA1430 (6.5 μM) by CAase (13.0 μM). No increase in A_610_ was observed in EPS-only or CAase-only controls over the time course; these values were used for background subtraction. Data in both panels are means ± SD (n  = 3).

The prolonged enzymatic activity of CAase is consistent with observations from other glycoside hydrolases. For instance, the enzyme WceF, which degrades the stewartan polysaccharide in *Pantoea stewartii*, retained measurable activity over multiday incubations due to its structural stability under assay conditions.^24^ These results suggest that CAase exhibits comparable structural and thermal stability, enabling continuous processing of complex EPS substrates with differing compositions (Tables 1 and 2).

**Table 1:** Relative Percentages of Glycosyl Residues Detected in EA273 Samples with and without CAase Treatment after 200 h^a^

EA273	No Treatment	CAase Treatment
Glycosyl residue	(Relative % Area)	(Relative % Area)
3-Galactopyranosyl residue (3-Gal)	25.2	15.6
4-Glucopyranosyl residue (4-Glc)	9.5	26.5
Other Residues	65.4	58.1
Total	100.0	100.0

a Chromatograms of the PMAAs generated from EA273 ± CAase samples used for linkage analysis are provided in Supplemental Figures S1 and S2. A complete list of all detected glycosyl residues is available in Supplemental Table S1.

**Table 2:** Relative Percentages of Glycosyl Residues Detected in EA1430 Samples with and without CAase Treatment after 200 h^a^

EA1430	No Treatment	CAase Treatment
Glycosyl residue	(Relative % Area)	(Relative % Area)
2,1-Fructose +2,6-Fructose	54.3	51.7
1,2,6-Fructose	19.9	18.6
t-Fructose	15.9	15
Other Residues	10.0	14.8
Total	100.0	100.0

a Chromatograms of the PMAAs generated from EA1430 ± CAase samples used for linkage analysis are shown in Supplemental Figures S3 and S4. Values are normalized relative % GC–MS peak area and are intended for comparative structural interpretation (semi-quantitative). A complete list of all detected glycosyl residues is available in Supplemental Table S2.

Differences in reaction kinetics between EA1430 and EA273 EPS likely reflect structural variations, such as branching patterns or glycosidic linkage types, that influence enzyme affinity and cleavage rate.^25,26^ These structural features are examined further in the linkage analysis.

### Glycosidic Linkage Analysis Reveals Substrate-Specific Cleavage Patterns by CAase

To determine the structural basis of CAase activity against *E. amylovora* EPS, glycosidic linkages in EPS from strains EA273 and EA1430 before and after enzyme treatment were analyzed using GC–MS, which provides semiquantitative, primarily qualitative information on linkage composition.^27–30^

EPS from EA273 was enriched in galactopyranosyl residues, particularly 3-linked Gal (25.2%), 3,4-Gal (8.8%), and terminal Gal (6.3%), consistent with the structure of amylovoran, the key virulence-associated EPS in *E. amylovora*.^31^ In contrast, EPS from EA1430 was dominated by 2,1- and 2,6-linked fructofuranosyl residues (54.3%), characteristic of levan, a β-2,6-linked fructan-type polysaccharide containing β-2,1 branches.^25^

These compositional patterns align with previous reports that demonstrate EA273 is a high-amylovoran producer, whereas EA1430 synthesizes less amylovoran and instead accumulates levan-type polymers.^31^ This divergence in EPS structure likely accounts for the differential enzyme responses observed in the MBTH assay (Figure 1) and suggests that CAase cleaves distinct glycosidic linkages in each strain.

Following CAase treatment, EPS from EA273 showed a marked reduction in 3-Gal residues (from 25.2% to 15.6%), which is consistent with enzymatic cleavage of amylovoran’s backbone. Simultaneously, the relative abundance of 4-Glc increased from 9.5% to 26.5%, likely reflecting intermediate breakdown products or enhanced exposure of secondary glycosidic linkages. This is consistent with previous findings showing that linkage analysis reflects relative abundance, and reductions in one component can lead to apparent increases in others due to normalization effects during methylation analysis.^32^ In EPS from EA1430, the dominant levan-associated fructofuranosyl linkages (2,1-Fru +2,6-Fru) showed a slight decrease following CAase treatment (Table 2), while terminal and branched fructose linkages changed to a lesser extent. These small differences may reflect limited enzymatic effects; however, GC–MS methylation/PMAA linkage analysis is primarily qualitative and semiquantitative, as relative % peak areas are normalized and sensitive to derivatization and hydrolysis efficiencies.^30^

Comparable enzymatic activity has been described for phage tail-associated depolymerases, such as the *Erwinia* phage L1 enzyme DpoL1, which specifically cleaves amylovoran and enables phage infection of encapsulated *E. amylovora* cells.^33^ While DpoL1 demonstrates clear substrate specificity and functions in concert with phage infection, CAase is recombinantly produced and active as a stand-alone enzyme.

Although *E. amylovora* populations from a given region are often reported to be highly genetically homogeneous, genetic similarity does not necessarily imply uniformity in physiological traits such as EPS composition, capsule characteristics, or relative production of amylovoran vs levan.^34^ Indeed, prior work has shown that amylovoran production is strain dependent and correlates with virulence, and that structural differences in amylovoran can occur among isolates, supporting the expectation that EPS heterogeneity exists even within closely related *E. amylovora* populations.^35^ Accordingly, the strain-dependent activity observed here (Figure 1; Tables 1–2) is consistent with a model in which CAase efficacy may depend on EPS structure and linkage composition, and suggests that variable responses across field populations should be anticipated.

Future work will correlate EPS structural features with CAase sensitivity across diverse *E. amylovora* isolates, engineer or combine depolymerases with complementary substrate specificities, and develop formulations and deployment strategies that maintain efficacy across heterogeneous pathogen populations.^36^

### CAase Disrupts the EPS Matrix of *E. amylovora* Biofilms in SEM Imaging

Scanning electron microscopy revealed that CAase treatment caused extensive degradation of the extracellular polysaccharide (EPS) matrix in *E. amylovora* strain EA273 biofilms. Untreated cultures (Figure 2A and C) formed a dense, mucoid EPS network that enveloped individual bacteria and spanned between cells as long, fibrous bridges. At higher magnification, this network appeared as a thick, sheath-like layer encasing the bacterial surface. In contrast, CAase-treated samples (Figure 2B and D) displayed a dramatic loss of extracellular matrix. Bacterial rods were cleanly delineated with minimal surface debris, indicating near-complete degradation of the EPS/biofilm structure. Notably, the overall surface morphology of CAase-treated cells appeared smoother and less textured than untreated controls.

**Figure 2:**
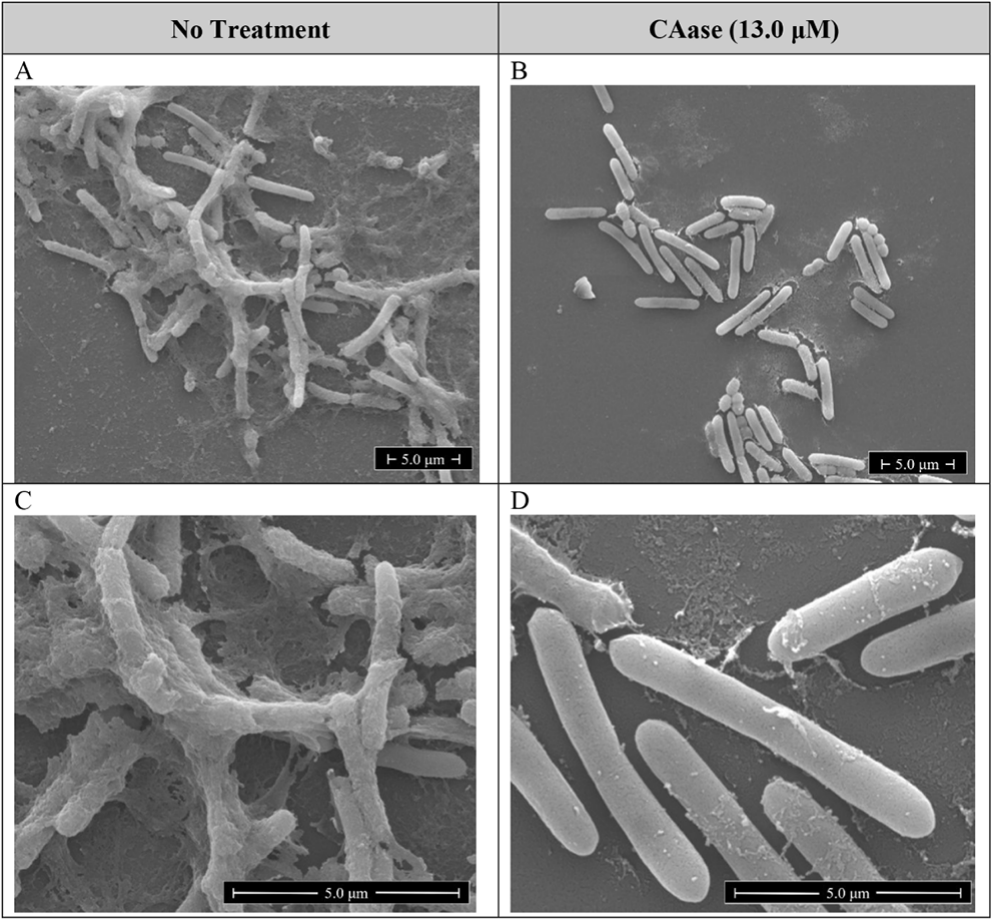
Scanning-electron micrographs illustrating the effect of CAase on the EPS matrix of *Erwinia amylovora* EA273 biofilms. Left-hand panels (A and C) show cells incubated 24 h in KB broth without enzyme. Right-hand panels (B and D) depict parallel cultures exposed for 24 h to 13.0 μM CAase. Scale bars: 2 μm (10,000 ×) and 5 μm (25,000 × ).

These observations are consistent with EPS degradation by CAase. While SEM does not directly measure catalysis, the loss of matrix aligns with reducing-end and linkage data. The disappearance of the extracellular coating after treatment supports the biochemical evidence from the MBTH assays and suggests that CAase can physically dismantle biofilm architecture and liberate cells from the matrix.

The smoother appearance of the treated cells further raises the possibility that CAase also modifies capsular or outer membrane-associated polysaccharides in addition to the extracellular material. Similar effects have been described for other glycoside hydrolases that act on capsular or surface-linked polymers.^37,38^ While the SEM images cannot conclusively establish membrane damage, the altered surface morphology suggests potential downstream impacts on envelope integrity which can be examined using membrane permeability assays or transmission electron microscopy.

### CAase Reduces Viability of *E. amylovora* in a Dose-Dependent Manner

To quantify the impact of CAase on *E. amylovora* viability, EA273 cells were enumerated following exposure to a range of increasing enzyme concentrations. A colony forming unit (CFU) assay was performed on strain EA273 following 18 h incubation with a range of CAase concentrations. The enzyme was tested at 0–20 μM in phosphate-buffered saline, and viable cell counts were quantified by serial dilution and spot plating on KB agar. The results revealed a dose–response relationship where the number of viable cells decreased with increasing enzyme concentrations (Figure 3).

**Figure 3:**
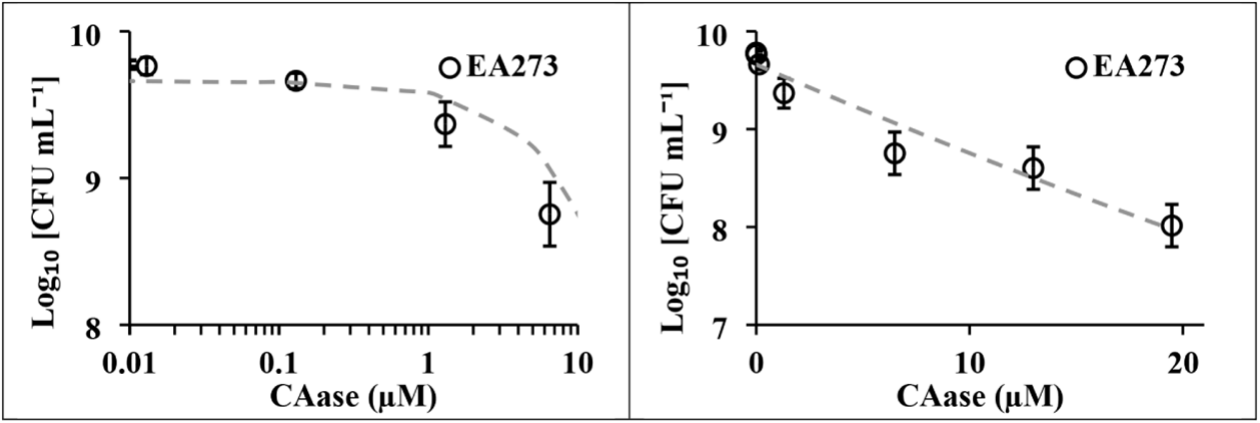
Dose–response relationship between CAase concentration and survival of *Erwinia amylovora* EA273. Left: Fine-scale titration of CAase (0.1–10 μM). Right: Expanded concentration range (0–20 μM). Lines represent exponential decay fits to the data. Points represent means ± SD (*n* = 3). PBS-treated samples served as controls.

Viability remained constant at CAase concentrations below 0.1 μM but a pronounced decline occurred near 1.3 μM, corresponding to approximately a 1-log reduction in CFU mL^–1^. At 20 μM CAase reduced the viable population by approximately 2 orders of magnitude. The data fit well to an exponential decay model, consistent with a concentration-dependent inhibitory effect. The minimum effective concentration (MEC) of ∼1.3 μM provides a useful benchmark for future formulation and application strategies. The bacteriostatic effect observed here is likely indirect; rather than lysing cells, CAase may alter nutrient gradients, disrupt quorum sensing, or increase susceptibility to desiccation and oxidative stress by eliminating the biofilm shield.^39^

These results indicate that CAase impairs *E. amylovora* viability in a concentration-dependent manner. Consistent with this interpretation, growth-curve analyses following a defined thermal challenge demonstrate that CAase pretreatment increases sensitivity to heat stress. As shown in Supplemental Figures S7 and S8, CAase-treated cells of strains EA273 and EA1430 exhibited markedly greater growth inhibition following exposure to heat stress compared with CAase treatment alone, whereas PBS-treated controls showed minimal differences between heated and nonheated conditions. Together, these data indicate that removal of the EPS matrix by CAase compromises the ability of *E. amylovora* to tolerate environmental stress. While CAase is not a conventional bactericide, disruption of the protective EPS matrix likely sensitizes cells to external stressors and interferes with biofilm-dependent survival mechanisms, consistent with prior studies demonstrating that enzymatic degradation of EPS increases bacterial susceptibility to antimicrobial agents or reduces metabolic resilience by eliminating the structural microenvironment that buffers stress.^39,40^

### Enzymatic Treatment with CAase Inhibits Surface Motility in *E. amylovora*

To determine whether CAase affects surface motility in *E. amylovora*, soft agar motility assays were performed using wild-type EA273. Cells were point-inoculated into 0.2% agar plates containing increasing concentrations of CAase (0.13–13.0 μM), and the area of bacterial spread was measured after incubation (Figure 4).

**Figure 4:**
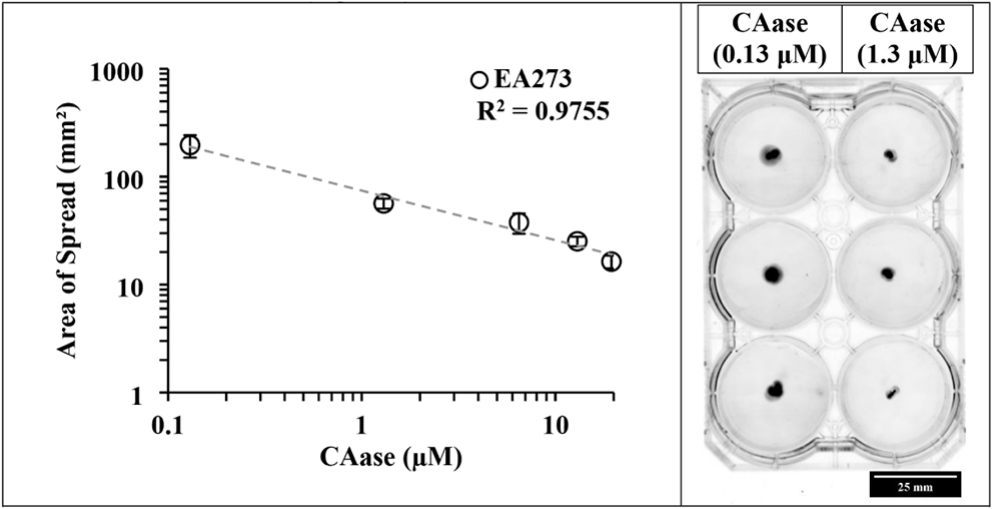
CAase inhibits surface motility of *E. amylovora* EA273 in soft agar. Left: Wild-type EA273 was point-inoculated into 0.2% agar plates supplemented with increasing concentrations of CAase (0.13–13.0 μM). Right: Representative soft agar motility plates of EA273 point-inoculated at the center.

A clear, dose-dependent decline in surface motility was observed with increasing CAase concentration. At 0.13 μM, *Erwinia amylovora* EA273 formed expansive, outward-spreading halos characteristic of active surface movement, whereas progressively higher CAase concentrations produced smaller zones of spread, with motility strongly suppressed at ≥1.3 μM. When analyzed on logarithmic axes, the relationship between CAase concentration and motility followed a power-law trend, indicating a strong inverse dependence of surface spread on enzyme dose (area of spread (mm^2^) = 74.3 × [CAase]^−0.458^; R^2^ = 0.9927; Figure 4). Residuals from the log–log regression were normally distributed (Shapiro–Wilk test, p = 0.53), supporting the appropriateness of the model.

These results suggest that CAase significantly impairs surface-associated movement of *E. amylovora*, a phenotype closely linked to virulence and colonization in plants.^41^ The suppression of motility likely arises from enzymatic degradation of extracellular polysaccharides required for type IV pilus or flagella-mediated movement on semisolid surfaces.^37^ Alternatively, CAase may alter the local microenvironment such as reducing hydration or modifying surface tension through biofilm matrix degradation, thereby impeding coordinated migration.^42^ Reduced motility has been previously correlated with decreased virulence in *E. amylovora* and other Gram-negative pathogens.^41,43^ Therefore, the ability of CAase to suppress bacterial spread may contribute not only to reduced biofilm formation but also to attenuated infectivity, reinforcing its potential as a multitarget biocontrol agent.

### TEM Imaging Shows CAase-Induced Ultrastructural Damage to *E. amylovora*

To examine the impact of CAase on *E. amylovora* at the unicellular level, transmission electron microscopy (TEM) was used to visualize bacterial morphology following 24 h incubation with or without CAase (13.0 μM). Both whole-mount imaging (Panels A–B) and cross-sectional views of resin-embedded cells (Panels C–F) were obtained to assess changes in membrane integrity and intracellular organization (Figure 5).

**Figure 5:**
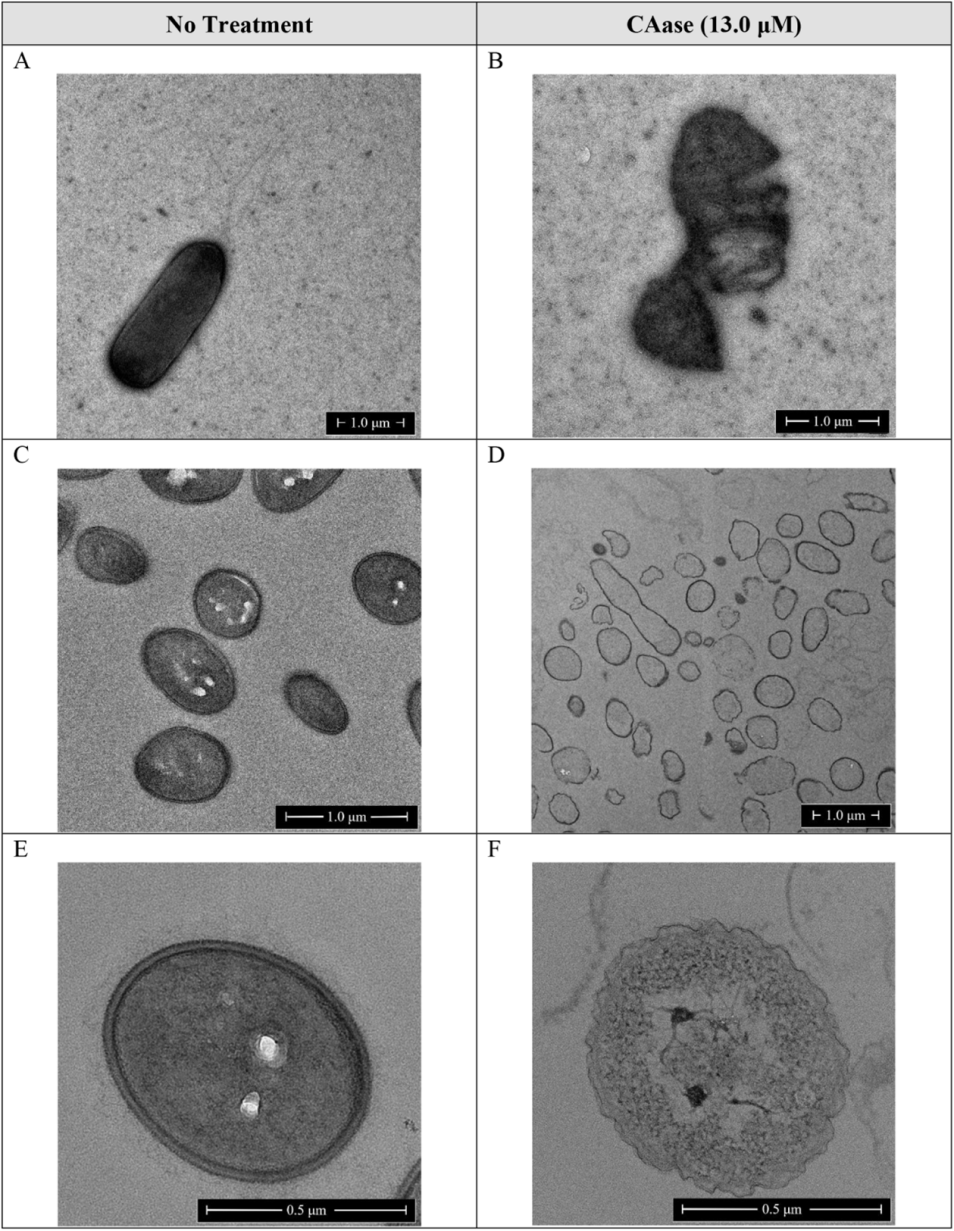
Transmission electron micrographs of *E. amylovora* EA273 cells treated with CAase. Panels A and B show whole-mount TEM images of untreated (A) and CAase-treated (B) cells acquired at 5,000× and 11,500× magnification, respectively. Panels C–F show cross-sectional TEM images of resin-embedded cells sectioned orthogonally, acquired at 14,500× (C), 5,000× (D), and 50,000× (E–F). Scale bars represent 1.0 μm in panels A–D and 0.5 μm in panels E–F.

In whole-mount micrographs, untreated cells (Panel A) displayed intact rod-shaped morphology, smooth outer membranes, and dense internal structure. In contrast, CAase-treated cells (Panel B) appeared distorted or collapsed, with irregular shapes and disrupted internal contents.

Cross-sectional TEM slices of resin-embedded cells (Panels C–F) further revealed structural differences. Control cells (Panels C and E) showed well-defined inner and outer membranes, clearly partitioned periplasmic space, and uniformly dense cytoplasm. By comparison, CAase-treated cells (Panels D and F) exhibited compromised envelope structure, with irregular or missing outer membranes, reduced cytoplasmic density, and evidence of lysis. Several cells appeared collapsed or displaced toward the center, which is consistent with reduced turgor pressure and compromised membrane integrity.

These structural alterations indicate that CAase treatment not only strips the biofilm matrix but also compromises envelope stability and cytoplasmic organization. While CAase’s primary target is extracellular EPS, its action may extend to surface-bound polysaccharides essential for maintaining envelope stability. Alternatively, the removal of the protective matrix may render cells more susceptible to osmotic stress, desiccation, or mechanical damage during growth and sample preparation.

Such outcomes are consistent with prior reports showing that enzymatic disruption of biofilms can lead to secondary damage to cell membranes, especially under prolonged exposure.^13^ These findings further support the hypothesis that CAase operates through both structural and antimicrobial mechanisms; dispersing biofilms while also impairing the physical integrity of pathogenic cells.

### CAase Reduces Blossom and Shoot Blight Incidence in Field Trials Compared to Untreated Control

Blossom blight and shoot blight represent the two most damaging phases of fire blight disease in apple orchards. Blossom blight occurs when *E. amylovora* colonizes flowers during bloom, leading to necrosis of blossoms and providing the initial infection sites for the pathogen.^44^ From these early infections, the bacterium often progresses into shoots, causing shoot blight, which manifests as characteristic tip wilting and dieback that can severely impact tree growth, productivity, and long-term orchard viability. Because blossom infections are the primary entry point and shoot blight reflects systemic spread and disease severity, both metrics are essential for evaluating the effectiveness of management strategies in field trials.^45^

In Year 1, at a low CAase concentration (1.82 × 10^–3^ μM), applications made once (1×) and twice (2×) provided 40% and 25% control of blossom blight, respectively (Figure 6). The absence of statistical separation among some treatments reflects intertree variability typical of orchard trials rather than identical mean treatment performance. In Year 2, a higher CAase concentration (13.0 μM) applied once (1×) and twice (2×) provided 54% and 66% control of blossom blight (Figure 7). Control of shoot blight, which follows the onset of blossom blight, was consistently higher, ranging from 55–75% across all CAase treatments and both years (Figures 6 and 7).

**Figure 6:**
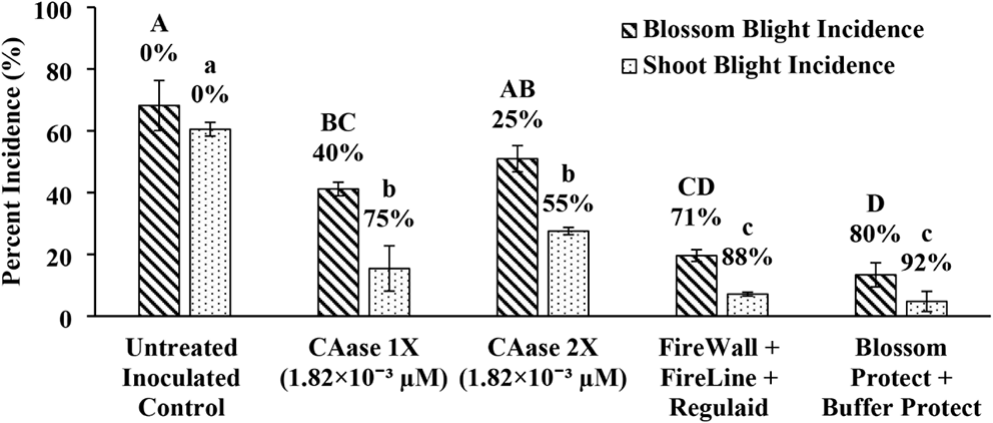
CAase Reduces Blossom and Shoot Blight Incidence in Field Trials at Low Concentration (1.82 × 10^–3^μM). “Fuji” apple trees treated with CAase at (1.82 × 10^–3^ μM) demonstrated significant reductions in blossom and shoot blight incidence compared to untreated controls. The effectiveness of CAase treatments was evaluated against standard antibiotic (FireWall + FireLine + Regulaid) and biological treatments (Blossom Protect + Buffer Protect). This trial was conducted during the 2023 growing season in Winchester, Virginia, USA. Percent control values shown above each bar are calculated relative to the untreated control (eq 2). Bars represent means of four single-tree replicates; error bars denote standard error of the mean. Bars sharing a letter are not significantly different; bars with different letters differ significantly (one-way ANOVA followed by Tukey’s HSD, α = 0.05). Capital letters denote statistical groupings for blossom blight, whereas lowercase letters denote statistical groupings for shoot blight.

**Figure 7:**
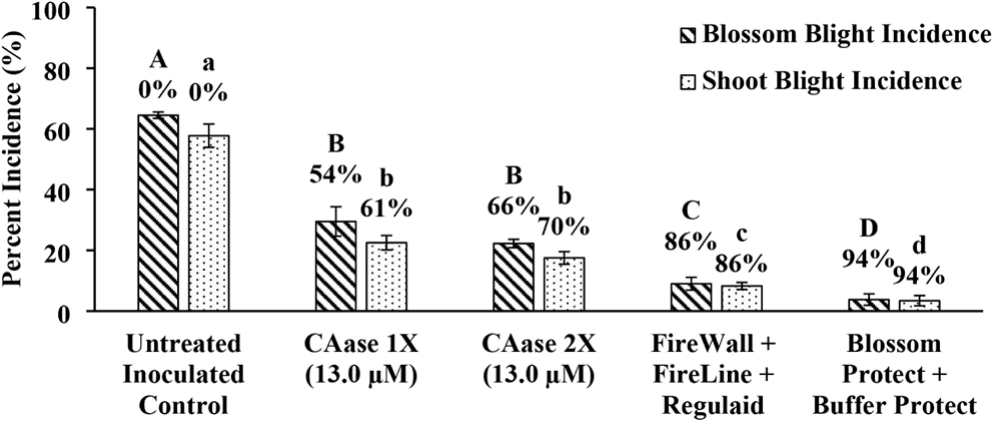
Higher Concentration of CAase (13.0 μM) Enhances Control of Blossom and Shoot Blight. Field trials using CAase at a higher concentration (13.0 μM). CAase treatments were assessed alongside a standard antibiotic treatment (FireWall + FireLine + Regulaid) and a biological control treatment (Blossom Protect + Buffer Protect). This trial was conducted during the 2024 growing season in Winchester, Virginia, USA. Percent control values shown above each bar are calculated relative to the untreated control (eq 2). Bars represent means of four single-tree replicates; error bars denote standard error of the mean. Bars sharing a letter are not significantly different; bars with different letters differ significantly (one-way ANOVA followed by Tukey’s HSD, α = 0.05). Capital letters denote statistical groupings for blossom blight, whereas lowercase letters denote statistical groupings for shoot blight.

The stronger relative suppression of shoot blight compared to blossom blight is consistent with the epidemiology of fire blight disease progression. Blossom blight represents the primary infection phase, during which *E. amylovora* populations establish and amplify on floral stigmas before invading nectaries and internal tissues. Infected blossoms subsequently serve as the dominant source of secondary inoculum, which is disseminated by rain and insects to initiate shoot blight infections.^44,45^ As a result, partial suppression of blossom infection can create a population bottleneck that limits subsequent shoot colonization, even when blossom blight control is incomplete. This pattern is well documented in fire blight management, where shoot blight incidence is often lower than blossom blight incidence following bloom-targeted interventions, including streptomycin-based programs that typically achieve high but incomplete control.^2,44^

In addition, shoots may experience enhanced protection due to greater surface area and architectural complexity, which can improve retention of applied materials relative to delicate floral tissues. Once established, shoot blight progression depends heavily on amylovoran-mediated biofilm formation and vascular colonization, processes expected to be antagonized by CAase through enzymatic degradation of EPS.^46^ These epidemiological and biological factors provide a mechanistic basis for the disproportionately stronger suppression of shoot blight observed following bloom-stage CAase applications.

CAase did not have a significant effect on fruit russeting, a disorder previously associated with *Aureobasidium pullulans*-based biological treatments.^47^ Consistent with this, CAase-treated fruit did not differ significantly from standard antibiotic treatments with respect to russeting in either year (Supplemental Figures S5 and S6).

The improved disease suppression observed at higher CAase concentrations in Year 2 suggests that dose optimization may partially compensate for reduced enzymatic efficiency against structurally distinct EPS substrates. Similarly, repeated applications during bloom may help maintain effective enzyme coverage during periods of rapid pathogen population expansion. Additionally, CAase may be most effective when deployed as a biofilm-disrupting adjuvant rather than as a universal standalone bactericide, particularly in combination with standard antibiotics, biological controls, or complementary enzymes targeting alternative EPS structures. Such integrated strategies may broaden efficacy across heterogeneous pathogen populations and represent a practical pathway for translating enzymatic EPS degradation into robust orchard-scale disease management.

## Formulas and Equations

1 $$CFU\;mL^{-1}=\frac{mean\;colony\;count}{v}\times 10^{d}$$

Where *mean colony count* is the average number of colonies counted on the plate or spot(s); *v* is the plating volume in milliliters used for that count (e.g., *v* = 0.005 mL for a 5 μL drop); and *d* is the absolute value of the base-10 dilution exponent of the plated sample (e.g., for a 10^–5^ plate, *d* = 5). The factor *10* ^*d*^ corrects for the dilution, so the final units are colony-forming units per milliliter (*CFU* mL^–1^).

2 $$\mathrm{Percent}\;\mathrm{Control}\;\left[\%\right]=\left|\frac{Blight\;Incidenc{e\;}_{Treatment}}{Blight\;Incidenc{e\;}_{Untreated}}-1\right|\times 100$$

Where *Blight Incidence_Treatment_* is the percentage of blossoms or shoots exhibiting fire blight symptoms following a given treatment, and *Blight Incidence_U__nt_ _reated_* is the corresponding percentage observed in untreated control trees. Percent control represents the relative reduction in disease incidence attributable to the treatment and was calculated independently for blossom blight and shoot blight using the same formulation. Multiplication by 100 converts the value to percent control relative to the untreated control.

## Supplementary Material



## Notes

Figures S1–S4: GC–MS chromatograms of partially methylated alditol acetate (PMAA) derivatives from EPS isolated from *Erwinia amylovora* strains EA273 and EA1430, with and without CAase treatment. Table S1 and S2: relative molar percentages of detected glycosyl residues from EPS of EA273 and EA1430, with and without CAase treatment, as determined by GC–MS linkage analysis; Figure S5 and S6: observed incidence of apple russeting during the 2023 and 2024 growing seasons following field treatments; Figure S7 and S8: Growth-curve analyses of *E. amylovora* strains EA273 (Figure S7) and EA1430 (Figure S8) following CAase pretreatment and subsequent heat stress (PDF)
