# Supplementary material for: Enzymatic Biocontrol of Fire Blight (Erwinia amylovora) Using an Engineered Glycosyl Hydrolase
Source: Environ Sci Technol. 2026 May 9;60(20):14636–46. doi: 10.1021/acs.est.5c16698 (PMC13217557; doi:10.1021/acs.est.5c16698)
Supplement: Supplementary file 1 [file es5c16698_si_001.pdf]

# Enzymatic Biocontrol of Fire Blight (*Erwinia amylovora*) Using an Engineered Glycosyl Hydrolase

**Authors**
Kevin J. Lynn[1], Cole Clapper[1], Evan Kulp[1], Nathanial J. Boeckman[2], Matheus C. Borba[2], Emmanuel Sempeles[2], Joseph Capobianco[3], Srđan Aćimović[2]*, Bryan W. Berger[1]*
[1]University of Virginia, Charlottesville VA
[2]Virginia Polytechnic Institute and State University, Blacksburg VA
[3]Characterization and Interventions for Foodborne Pathogens, Agricultural Research Service, United States Department of Agriculture, Wyndmoor, PA
*Corresponding authors: B.W.B., bwb2k@virginia.edu, S.G.A. acimovic@vt.edu

**Summary**

The Supporting Information contains eight figures and two tables that provide additional analytical and experimental detail supporting the main findings. Figures S1–S4 present GC–MS total ion chromatograms of partially methylated alditol acetates (PMAAs) used for glycosyl linkage analysis of *Erwinia amylovora* strains EA273 and EA1430, with and without CAase treatment. Figures S5 and S6 summarize fruit russeting outcomes from 2023 and 2024 field trials, demonstrating that CAase treatment does not increase russet incidence relative to untreated or antibiotic-treated controls, in contrast to Blossom Protect. Figures S7 and S8 show growth-curve analyses indicating that CAase pretreatment increases heat-stress sensitivity in both strains. Tables S1 and S2 report the relative abundance of detected glycosyl residues for EA273 and EA1430, respectively, highlighting strain-dependent differences in exopolysaccharide composition and the impact of CAase treatment on glycosyl linkage profiles.

**Supporting Information**

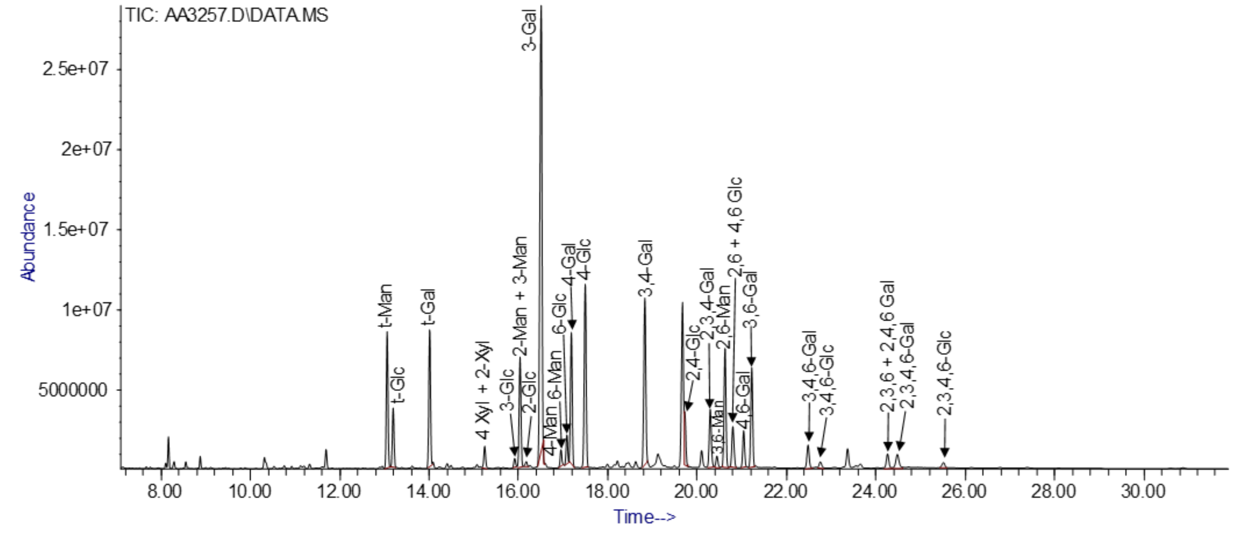


**Figure S1.** GC-MS (TIC) chromatogram of the PMAAs generated from the EA273 sample used for linkage analysis

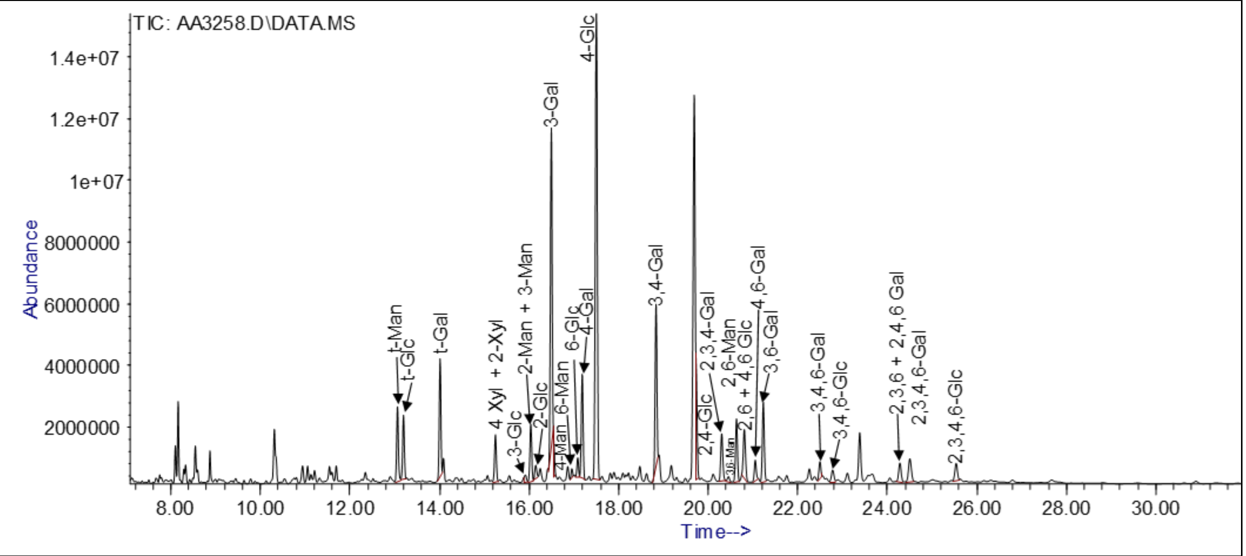


**Figure S2.** GC-MS (TIC) chromatogram of the PMAAs generated from the EA273+CAase sample used for linkage analysis

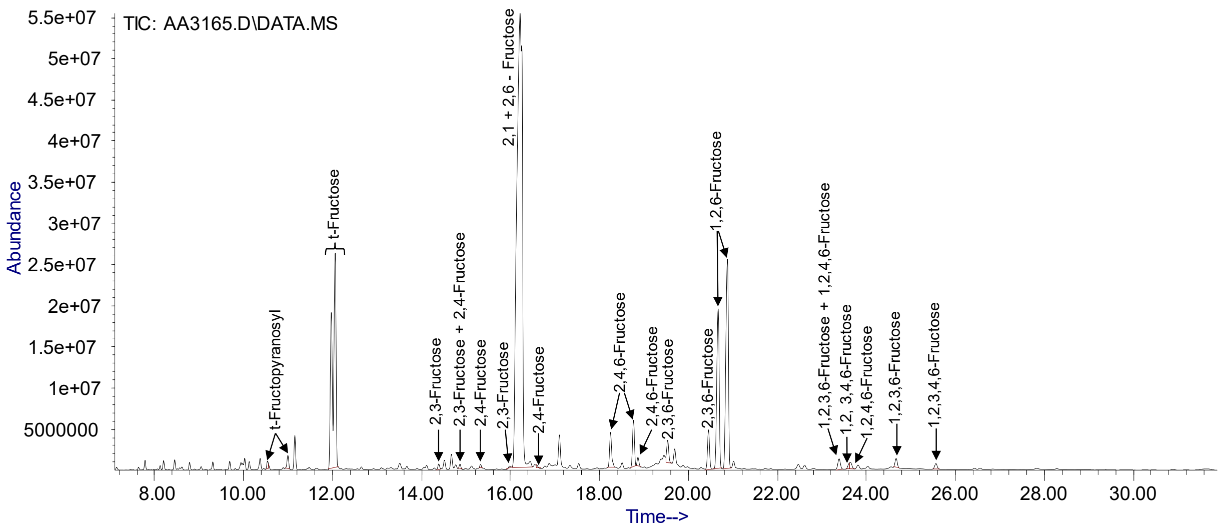


**Figure S3.** GC-MS Total Ion Chromatogram (TIC) chromatogram of the PMAAs generated from the EA1430 sample used for linkage analysis

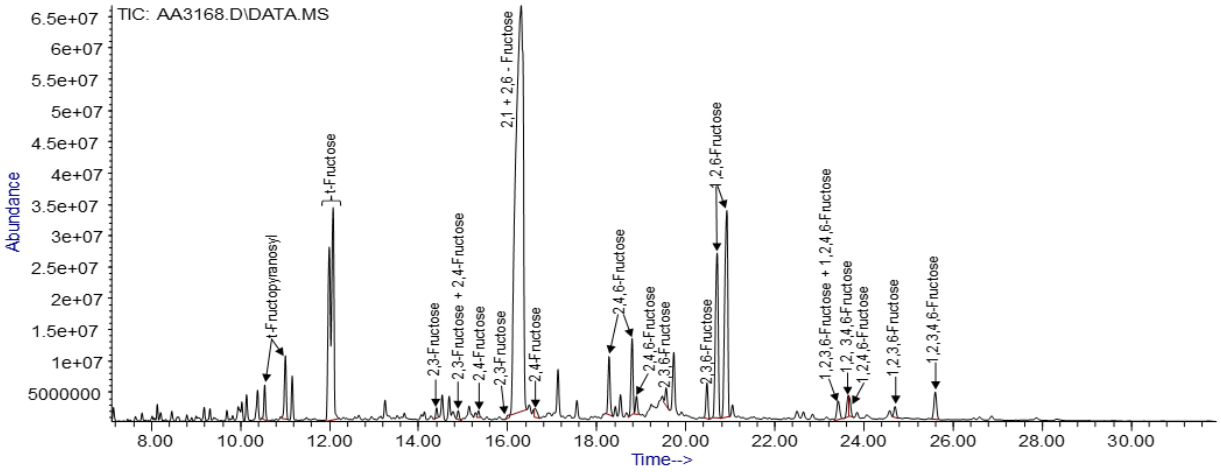


**Figure S4.** GC-MS (TIC) chromatogram of the PMAAs generated from the EA1430+CAase sample used for linkage analysis

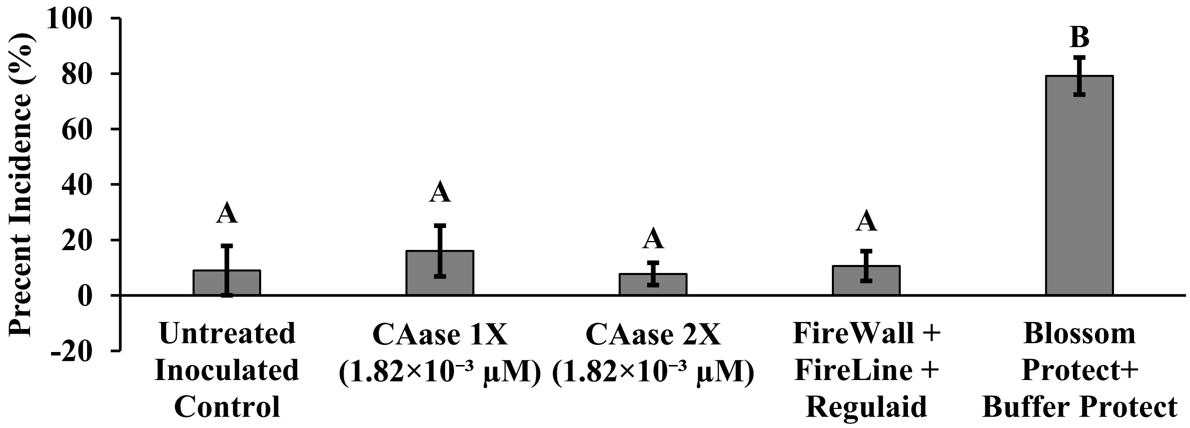

**Figure S5. CAase Does Not Induce Fruit Russeting Unlike Blossom Protect 2023.** Russet incidence was assessed 73 days post-inoculation on fruit clusters from trees treated with CAase (1X and 2X at $1.82\times10^{-3}$ µM)), FireWall + FireLine + Regulaid, and Blossom Protect + Buffer Protect. CAase and antibiotic treatments did not significantly increase russeting compared to the untreated inoculated control, while Blossom Protect resulted in a significantly higher russet incidence. Bars labeled with different letters indicate statistically significant differences (ANOVA with Tukey's HSD test, $\alpha = 0.05$). Error bars represent standard error of the mean.

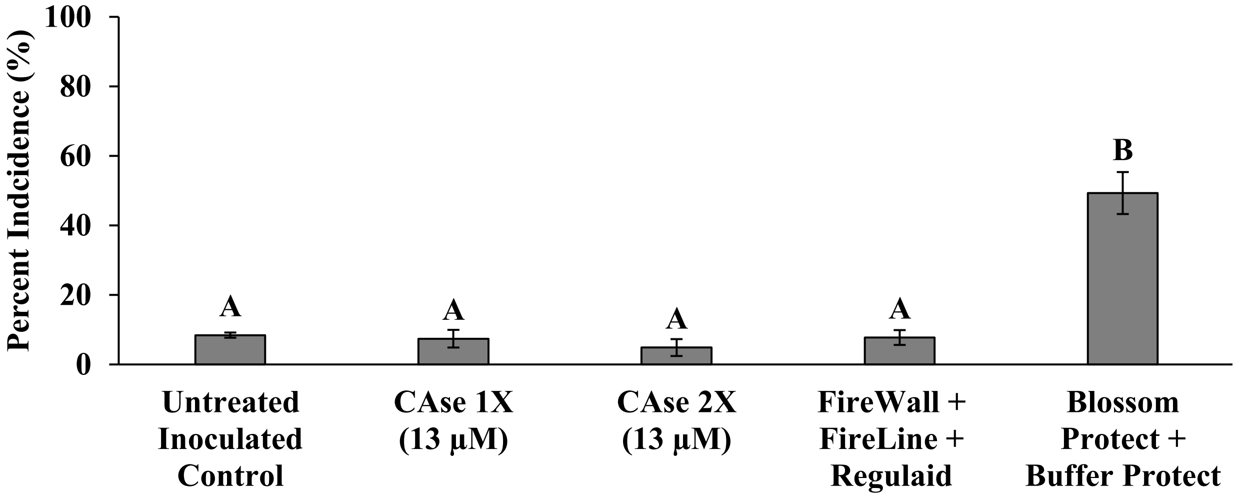


**Figure S6. CAase Does Not Induce Fruit Russeting Unlike Blossom Protect 2024.** Russet incidence was assessed 72 days post-inoculation on fruit clusters from trees treated with CAase (1X and 2X at 13 µM)), FireWall + FireLine + Regulaid, and Blossom Protect + Buffer Protect. CAase and antibiotic treatments did not significantly increase russeting compared to the untreated inoculated control, while Blossom Protect resulted in a significantly higher russet incidence. Bars labeled with different letters indicate statistically significant differences (ANOVA with Tukey's HSD test, $\alpha = 0.05$). Error bars represent standard error of the mean.

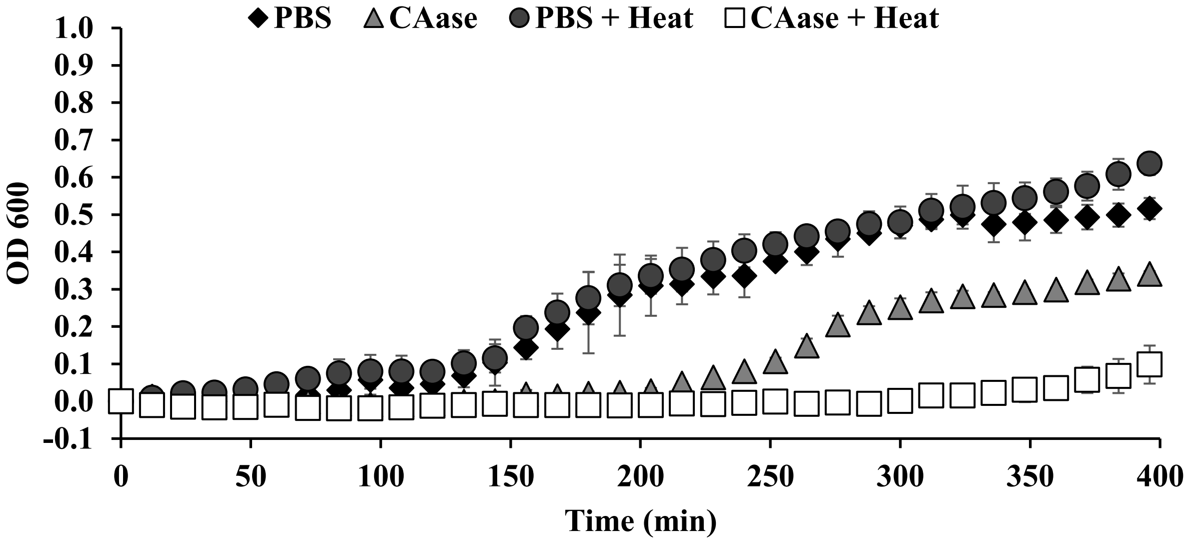


**Figure S7: CAase pretreatment increases heat-stress sensitivity in *Erwinia amylovora* EA273.** Growth-curve analysis of *E. amylovora* strain EA273 following CAase pretreatment and subsequent heat stress. Cultures were treated with CAase (13 µM) or PBS (negative control) for 12 h, followed by exposure to heat stress (42 °C for 25 min) or no heat stress. Data are shown as mean ± SD.

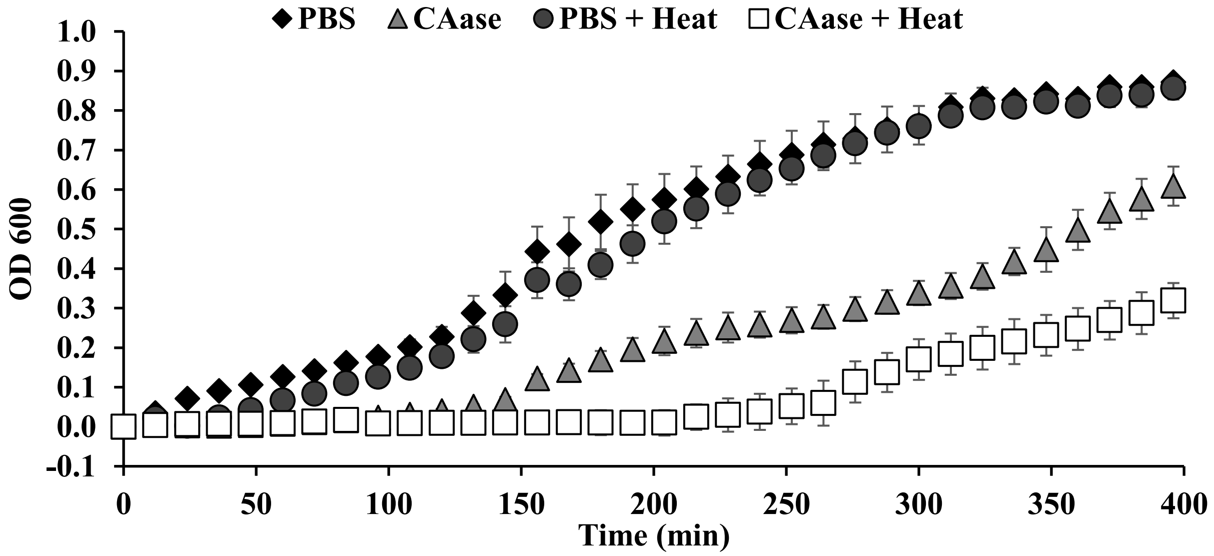


**Figure S8: CAase pretreatment increases heat-stress sensitivity in *Erwinia amylovora* EA1430.**

Growth-curve analysis of *E. amylovora* strain EA1430 following CAase pretreatment and subsequent heat stress. Cultures were treated with CAase (13 µM) or PBS (negative control) for 12 h, followed by exposure to heat stress (42 °C for 25 min) or no heat stress. Data are shown as mean ± SD.

**Table S1.** Relative percentage of each detected glycosyl residue in the EA273 and EA273 + CAase samples.

| EA273<br>Glycosyl residue | No Treatment<br>(Relative % Area) | CAase Treatment<br>(Relative % Area) |
|---|---|---|
| 3-Galactopyranosyl residue (3-Gal) | 25.2 | 15.6 |
| 4-Glucopyranosyl residue (4-Glc) | 9.5 | 26.5 |
| 3,4-Galactopyranosyl residue (3,4-Gal) | 8.8 | 8.2 |
| 4-Galactopyranosyl residue (4-Gal) | 6.4 | 5.3 |
| t-Galactopyranosyl residue (t-Gal) | 6.3 | 5.4 |
| 2,6-Mannopyranosyl residue (2,6-Man) | 6.2 | 3.4 |
| t-Mannopyranosyl residue (t-Man) | 6.1 | 3.5 |
| 2 + 3-Mannopyranosyl residue (2 + 3-Man) | 5.3 | 2.9 |
| 3,6-Galactopyranosyl residue (3,6-Gal) | 5.1 | 4.2 |
| 2,3,4-Galactopyranosyl residue (2,3,4-Gal) | 3.3 | 2.8 |
| t-Glucopyranosyl residue (t-Glc) | 2.7 | 2.9 |
| 2,6 + 4,6-Glucopyranosyl residue (2,6 + 4,6-Glc) | 2.4 | 2.9 |
| 2,4-Glucopyranosyl residue (2,4-Glc) | 2.3 | 3.7 |
| 4,6-Galactopyranosyl residue (4,6-Gal) | 1.8 | 1 |
| 3,4,6-Galactopyranosyl residue (3,4,6-Gal) | 1.4 | 0.8 |
| 6-Glucopyranosyl residue (6-Glc) | 1.3 | 0.9 |
| 2,3,4,6-Galactopyranosyl residue (2,3,4,6-Gal) | 1.1 | 1.8 |
| 4-Xylopyranosyl + 2-Xylopyranosyl residue (4 Xyl + 2-Xyl) | 1 | 2.3 |
| 2,3,4 + 2,4,6 Galactopyranosyl residue (2,3,6 + 2,4,6 Gal) | 0.9 | 1.3 |
| 6-Mannopyranosyl residue (6-Man) | 0.7 | 0.4 |
| 3,6-Mannopyranosyl residue (3,6-Man) | 0.6 | 0.2 |
| 3-Glucopyranosyl residue (3-Glc) | 0.4 | 0.4 |
| 3,4,6-Glucopyranosyl residue (3,4,6-Glc) | 0.4 | 0.8 |
| 2,3,4,6-Glucopyranosyl residue (2,3,4,6-Glc) | 0.4 | 1.2 |
| 2-Glucopyranosyl residue (2-Glc) | 0.3 | 0.7 |
| 4-Mannopyranosyl residue (4-Man) | 0.2 | 1.1 |
| **Total** | **100.0** | **100.0** |

**Table S2:** Relative percentage of each detected glycosyl residue in the EA1430 and EA1430 + CAase samples.

| EA1430 | No Treatment | CAase Treatment |
|---|---|---|
| **Glycosyl residue** | **(Relative % Area)** | **(Relative % Area)** |
| 2,1-Fructose + 2,6-Fructose | 54.3 | 51.7 |
| 1,2,6-Fructose | 19.9 | 18.6 |
| t-Fructose | 15.9 | 15 |
| 2,4,6-Fructose | 3.8 | 5.1 |
| 2,3,6-Fructose | 2.7 | 1.7 |
| t-Fructopyranosyl | 0.8 | 2.9 |
| 1,2,3,6-Fructose + 1,2,4,6-Fructose# | 0.6 | 0.9 |
| 1,2,3,6-Fructose | 0.5 | 0.4 |
| 1,2,3,4,6-Fructose | 0.5 | 2 |
| 2,3-Fructose | 0.3 | 0.4 |
| 2,4-Fructose | 0.3 | 0.6 |
| 1,2,4,6-Fructose | 0.3 | 0.5 |
| 2,3-Fructose + 2,4-Fructose# | 0.2 | 0.3 |
| **Total** | **100.0** | **100.0** |

*: >98% 2,6-Fructose; #: merged peaks
